# CO_2_ Elevation and Photoperiods North of Seed Origin Change Autumn and Spring Phenology as Well as Cold Hardiness in Boreal White Birch

**DOI:** 10.3389/fpls.2020.00506

**Published:** 2020-04-28

**Authors:** Binyam Tedla, Qing-Lai Dang, Sahari Inoue

**Affiliations:** ^1^Faculty of Natural Resources Management, Lakehead University, Thunder Bay, ON, Canada; ^2^Centre for Boreal Research, Northern Alberta Institute of Technology, Peace River, AB, Canada

**Keywords:** tree migration, climate change, elevated [CO_2_], photoperiod, white birch, leaf senescence, cold hardiness, phenology

## Abstract

The distribution of tree species is expected to shift toward the pole in response to the climate change associated with the elevation of atmospheric CO_2_ concentration [CO_2_]. The shift will expose trees to a new photoperiod regime and other environmental conditions. The changes in these factors will likely have interactive effects on the ecophysiological traits of plants. This study investigated how CO_2_ elevation and change in photoperiod influence the timing of bud development, leaf senescence, and cold hardiness in the fall, and bud break in the spring in boreal white birch (*Betula papyrifera* Marsh.). Seedlings were exposed to two different [CO_2_] (AC = 400 μmol mol^–1^; EC = 1000 μmol mol^–1^) and four simulated photoperiod regimes in the greenhouse corresponding to each latitude [48 (seed origin), 52, 55, and 58°N] for two growing seasons. We found that EC advanced the initiation of leaf color change (10% leaf color change) in the fall by 23 days, but delayed the completion date of color change (90%). Leaf senescence started earlier in the photoperiods corresponding to 55 and 58°N latitude than those at 48 and 52°N latitudes under EC, but photoperiod did not affect leaf senescence under AC. Additionally, the temperature causing 50% electrolyte leakage (a measure of susceptibility to freezing damage) was more negative under the photoperiod corresponding to 55° (−46°C) and at 58°N (−60°C) under EC than at the lower latitudes (above −40°C). Budburst in the spring occurred earlier under the photoperiods corresponding to the two highest latitudes under EC, but the trend was opposite under AC. The combination of longer photoperiods and elevated [CO_2_] resulted in earlier budburst in the spring and later completion of leaf senescence in the fall as well as greater cold hardiness, leading to extended growing seasons from both ends. However, the onset of leaf senescence was earlier than in other treatment combinations. Furthermore, the photoperiod effects were quite different under the ambient [CO_2_]. Our results suggest that it is extremely important to consider the complex interactions of [CO_2_] and photoperiod in planning latitudinal seed transfers and in predicting the migration of boreal trees in response to climate change.

## Introduction

Trees in the temperate and boreal regions experience seasonal growth cessation and dormancy which allow them to withstand the adverse climate condition in the winter. The timing of the phenological events, such as growth cessation, bud set, leaf senescence, development of cold tolerance, and budburst, is a major factor influencing the fitness, survival, and geographic distribution of the species (Chuine, 2010; Richardson et al., 2013). The key environmental drivers influencing the timing of those phenological events have attracted considerable attention in the research community (Heide, 1993a, b; Korner and Basler, 2010). The phenological responses of trees to climate change have been studied extensively (Korner and Basler, 2010; Fu et al., 2014). However, the potential changes in tree phenology associated with climate change-induced migration are still poorly understood (Way and Montgomery, 2015), for instance, we still do not fully understand how the changes in photoperiod regime associated with northward migration will affect the timing of phenology, fitness, survival, and growth of boreal trees (Way and Montgomery, 2015). Generally, when a tree species or genotype moves to a higher latitude from their current location, they will be exposed to longer photoperiods in the growing season and faster rates of change in photoperiod during the transition between growing season and non-growing season (Thomas and Vince-Prue, 1997). Both the change in photoperiod and the rate of change can alter the timing and duration of phenological events (Li et al., 2003). The threshold photoperiods for triggering autumn phenological events generally occur later when a species or genotype moves to higher latitude and the change in the timing can upset the harmony between the physiological state of the tree and the local climate conditions, which can cause several damages to trees by “unseasonal” environmental stresses, such as early frosts (Velling, 1979; Way and Montgomery, 2015).

Bud dormancy is a required growth transition consisting of a sequence of events such as the termination of apical elongation, the development of resting buds, an increase in frost tolerance and leaf senescence (Thomas and Vince-Prue, 1997). Once chilling requirements fulfilled, dormancy is released to permit growth resumption when the environmental conditions become favorable. Tree species with a wide geographic range of distribution generally have evolved mechanisms to trigger phenological changes and dormancy induction when the photoperiod reaches a critical level (Howe et al., 1995; Newaz et al., 2016; Inoue et al., 2019). The critical photoperiod that triggers the shift from active growth to cold acclimation has been found to increase with latitude in *B. papyrifera* (Downs and Bevington, 1981), *B. pendula* (Li et al., 2003), and some other tree species (Howe et al., 1995). The rate of dormancy development and cold acclimation can be affected by the rate of change in photoperiod (Howe et al., 2003; Chen et al., 2012). While the shorter critical photoperiod for triggering autumn phenology in trees adapted to a lower latitude allows them to have a longer growing season at a site of higher latitude (Thomas and Vince-Prue, 1997), it will also lead to a shorter period of time for the development of cold hardiness and nutrient resorption during leaf senescence (Way and Montgomery, 2015). For example, when a provenance of *B. pedula* is grown in a nursery at a higher latitude than that of the seed origin, the growth cessation in the fall occurs later, nutrient resorption is less complete and cold hardiness is less, leading to a high rate of mortality when the seedlings are planted in the field (Raulo, 1976; Velling, 1979). Longer photoperiods can also lead to earlier budburst in the spring, subjecting the trees to damages by spring frosts (Newaz et al., 2016). Furthermore, photoperiod can interact with other environmental factors in influencing the development of dormancy and cold hardiness (Li et al., 2015; Inoue et al., 2019; Tedla et al., 2020).

Elevated CO_2_ can also affect bud phenology (Murray et al., 1994), cold hardiness (Jach et al., 2001), and leaf senescence (Taylor et al., 2008). The response in the timing of spring bud break to elevated CO_2_ varies with species, ranging from advancing (Repo et al., 1996), to delaying (Murray et al., 1994), to no response (Norby et al., 2003). CO_2_ elevation can also advance the timing of bud set, increase cold hardiness (Murray et al., 1994) or decrease cold hardiness (Margolis and Vézina, 1990). Growing under elevated [CO_2_] can also advance leaf senescence in some species (Körner et al., 2005) but delay leaf senescence in other species (Körner et al., 2005) or have no effect in still other species (Herrick and Thomas, 2003). The wide variations in response to elevated [CO_2_] among tree species further complicate the prediction of interactive effects of elevated [CO_2_] and photoperiod regimes on trees. A good understanding of such interactive effects on the phenology and physiology of trees is critically important in the context of northward migration under a changing climate.

White birch (*Betula papyrifera* Marsh.) is a widely distributed birch species in North America. Generally, *Betula* spp. is known to have developed ecotypes that are adapted to the local photoperiod regime and have different photoperiods for growth cessation, dormancy induction, and cold acclimation (Li et al., 2003). This study investigates how changes in the photoperiod regime associated with climate change-induced northward migration influence the phenological responses of white birch to CO_2_ elevation. We hypothesize (1) that the faster rate of change in photoperiod at a higher latitude during the transition between growing and the non-growing season will negatively impact the development of cold hardiness in the fall, (2) that the later arrival of the threshold photoperiod for triggering a phenological shift in the fall at a higher latitude will delay the development of resting buds and leaf senescence, and (3) that CO_2_ elevation will alter the phenological response to photoperiod.

## Materials and Methods

### Plant Material

Mature seed catkins of white birch (*B. papyrifera* Marsh) were collected from 12 natural trees in Thunder Bay (48.4215°N, 89.2619°W) in September 2016. Seeds were extracted manually, air dried and stored in plastic bags at room temperature. The seeds from all the trees were mixed. The experiment was conducted at the Lakehead University Forest Ecology Complex in Thunder Bay. The seeds were sown in germination trays (50 × 25 cm) filled with a 1:1 (v:v) peat moss and vermiculite mixture. The day/night temperature and photoperiod during germination were set to 22/16°C and 16 h, respectively. Seedlings of relatively uniform size (2 cm average height) were transplanted into pots of 12 cm deep and 12/9.5 cm top/bottom diameter for the experiment. The growing medium was a mixture of vermiculite and peat moss (1:3, v:v).

### Experimental Design

The experiment was carried out as a split-plot design. The whole plot was CO_2_ concentration (AC 400 vs. EC 1000 μmol mol^–1^). The ambient [CO_2_] represents the latest reading at the time of the experiment at the Mauna Loa Observatory^1^. The EC is the IPCC projected CO_2_ concentration at the end of the century (Pachauri et al., 2014). Each CO_2_ level was replicated twice in two independent greenhouses. There were four photoperiod regimes (split-plot) within each whole plot corresponding to 48 (seed origin), 52, 55, and 58°N latitude. There were 15 seedlings within each treatment-replicate combination. A total of 240 seedlings (2 CO_2_ × 4 photoperiod × 2 replicate × 15 seedlings in each combination) were used for the study.

The CO_2_ elevation was achieved using natural gas CO_2_ generators (model GEN-2E; Custom Automated Products Inc., Riverside, CA, United States). The photoperiod regime in each greenhouse was set to emulate the longest of the four photoperiod treatments, and other photoperiods were obtained by shading the seedlings with black-out shade at the beginning and the end of each day. The photoperiod settings were adjusted weekly to emulate the seasonal change in photoperiod at the four latitudes. To facilitate the shading and to be consistent across all the treatments, a wooden frame was established around each split-plot. High-pressure sodium (HPS) lamps (P.L. Systems, Grimsby, ON, Canada) was used to extend the natural photoperiod when the natural day-length in the greenhouse was shorter than required. The HPS lamps provided about 600 μmol m^–2^ s^–1^ photosynthetically active radiation at the canopy level, with less blue light than sunlight but a similar R/FR ratio. The temperature in each greenhouse was set to emulate the daily temperature changes in the Thunder Bay region and ramped at 4 points (4:00, 10:00, 16:00, and 22:00). The temperature records of the past 10 years (2006–2016) were obtained from Environment Canada and the hourly averages were used as the set points. The seed origin temperature was used in the experiment with the assumption that tree species are expected to migrate poleward so that the populations remain in a similar climate as at their current location (McKenney et al., 2007, 2011). The [CO_2_], light, temperature, and humidity were all controlled and monitored using an Argus Titan Environment-control system (Argus Control Systems Ltd, Vancouver, BC, Canada). The photoperiod and temperature settings were adjusted weekly to emulate the natural seasonal pattern during the growing season. The experiment was carried out for two growing cycles. The first cycle was conducted between Nov. 24, 2016, and May 2, 2017, and the environmental conditions were set to emulate the natural environmental conditions of Jun. 7 to Nov. 15. The second cycle was between Nov. 1, 2017, and May 20, 2018, and the environmental conditions were set to emulate the natural conditions of April 26 to Nov. 12. The dates referred to hereafter are the emulated natural dates.

The volumetric water content of the growing medium was maintained above 50% of the field capacity as determined using an HH2 Moisture Meter and ML2X Theta Probe (DELTA-T DEVICES, Cambridge, United Kingdom). The seedlings were fertilized twice a week with a fertilizer solution containing 50/81/30.3 mg L^–1^ N/P/K from April 26 to May 25; 150/65.2/125 mg L^–1^ N/P/K from May 26 to August 30; and 50/54.3/156.3 mg L^–1^ N/P/K from 1–25 of September (Plant Products Co Ltd, Brampton, ON, Canada). There was no fertilizer application for the remainder of the growing season. At the end of the first growing season, the seedlings were stored in a cold store (-4°C) before the initiation of the second growing season. By the end of the growing season, the seedlings set buds and cold hardened. The seedlings were moved to cold storage and kept at -4°C. After 5 months of storage, the seedlings were transplanted into bigger pots (18 cm deep, 16/14 cm top/bottom diameter) and moved back to their original treatments to start the second growing season. The average seedling height and root collar diameter were 76 and 0.9 cm, respectively, at the end of the first growing season, and 178 and 1.6 cm, respectively, at the end of the second growing season.

### Observation of Bud Set and Leaf Senescence

A random sample of four seedlings per treatment combination (per replication) was used to monitor the timing of bud set and leaf senescence in August and September (emulated dates). The terminal bud development was divided into four stages: Stage 0- no terminal bud is visible; Stage 1 – bud visible for the first time; Stage 2 – bud scales are closed but are still green in color; and Stage 3 – bud-scales turn red-brown. Bud set was considered complete when the terminal bud reached stage 2. Mariën et al. (2019) have conducted an extensive comparison of different proxies to determine the timing of leaf senescence in deciduous trees and found that the measurement of chlorophyll degradation and the visual observation of leaf color change yield similar results. In this study, we determined the progress of leaf senescence by observing leaf color change every 5 days from August to November. We divided the progress into three stages: the initial stage when 10% of the leaves turned to yellow from green, intense coloration stage when 50% of the leaves changed color, and completion stage when 90% of the leaves changed color.

### Cold Hardiness Test

The cold hardiness was assessed after the completion of leaf senescence at the end of each growing season using the ion-leakage technique (Sukumaran and Weiser, 1972). The top 10 cm of the main stem was taken from four seedlings per treatment combination and cut into 2-cm sections. Each section was kept in a separate 50 mL Falcon centrifuge tube with 30 mL deionized distilled water overnight. The sections were taken out of the solution and frozen progressively at −5, −15, −30 and −45°C in a programmable freezer (Model 45–6.8; ScienTemp Corp., Adrian, MI, United States). The freezer temperature started from +5°C and was lowered at a rate of 5°C per hour and held for 1 h after each 5°C change. 1 h after the testing temperature was reached, the samples were removed from the freezer and thawed at room temperature overnight in 30 mL of deionized distilled water. The initial electrical conductivity of the solution was measured using an Accumet AR 20 electrical conductivity meter (Fisher Scientific, Ottawa, Canada). The samples were then placed in a drying-oven at 80°C for 2 h, and the electrical conductivity was measured again. The relative electrolyte leakage (REL) due to freezing damages was calculated as a ratio of the first and second measurements of the electrical conductivity. We used a linear interpolation of REL and freezing temperature relationship to estimate the lethal temperature that caused 50% electrolyte leakage (LT_50_).

### Timing of Bud Break

The progress of spring bud break was monitored on the same seedlings that were used for the observation of bud set and leaf senescence. Since the first growing season started with seeds, this observation was only conducted in the second growing season. For each seedling, we monitored five lateral buds adjacent to the terminal bud. The observations were made visually every 2 days, from May to June. The progress of bud break was divided into seven phases according to Linkosalo and Lechowicz (2006); 1 = the bud is completely closed; 2 = the bud starts to swell, 3 = the bud scales split open; 4 = the first leaf emerges; 5 = the base and petiole of the first leaf appear; 6 = all leaves completely emerge from the bud; and 7 = all leaves completely expand (i.e., reach full-size). The bud break of a seedling was considered complete when three or more buds reached phase 5.

### Statistical Analysis

The data were analyzed using two-way ANOVA with CO_2_ as the main plot and photoperiod as the sub-plot. Since the Shapiro test and residual plots showed that the data did not meet the ANOVA assumptions of normality and homogeneity, power transformation was conducted before ANOVA was carried out. Multiple comparisons were conducted when ANOVA showed a significant (*P* ≤ 0.05) interaction or significant photoperiod effect. Because the sample size in this study was relatively small and thus there was a high likelihood of making a type II error (i.e., failure to detect real treatment effects), Fisher’s LSD test was chosen for the multiple comparisons (Klockars and Sax, 1986). All the tests were conducted using the open-source statistical package *R* (Development Core Team 2018, V. 3.5.0, R).

## Results

### Bud Set and Leaf Senescence

**FIGURE 1:**
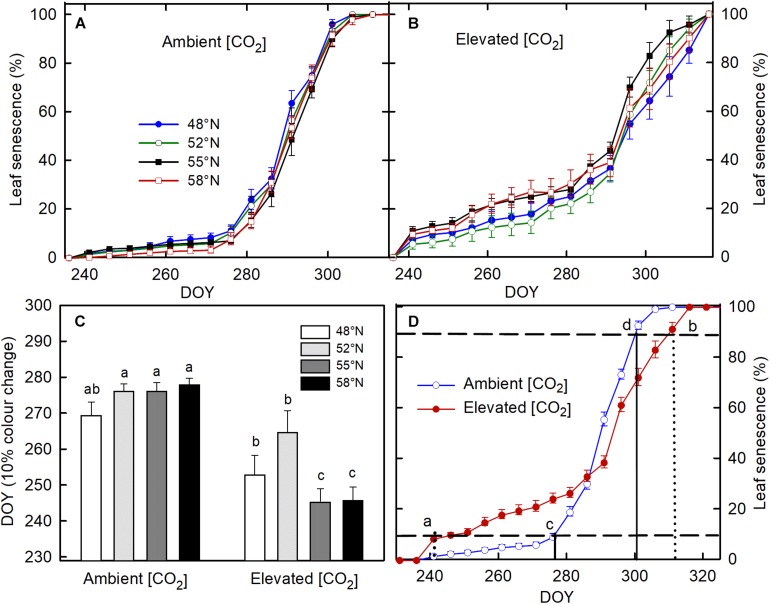
The effect of photoperiod regime on the progression of leaf senescence under ambient **(A)** and elevated (CO_2_; **B)**. **(C)** the interactive effect of CO_2_ and photoperiod on day of year (DOY) for 10% leaf color change with different letters within each CO_2_ and between ambient and elevated CO_2_ at a given photoperiod indicate significant difference from each other. **(D)** the progress of leaf color changes as affected by [CO_2_]. Seedlings were grown under photoperiod regimes corresponding to 48° (seed origin), 52°, 55°, and 58°N latitude and under 400 (ambient) and 1000 μmol mol^– 1^ (elevated) for two growing seasons. The vertical line a (dotted) and c (solid) in (**D**) denotes DOY for 10% leaf color change, under the elevated and ambient [CO_2_], respectively, while the vertical line b (dotted) and d (solid) represent DOY for 90% leaf color change at the elevated and ambient [CO_2_], respectively. The horizontal broken lines in (**C**) indicate 10 and 90% leaf color change. The value of each point represents mean ± SE of 8 (**A–C**) and 32 **(D)** seedlings.

None of the treatments significantly affect the timing of bud formation in either of the two growing seasons (Table 1). The bud set in all the treatment combinations was completed by 26 September (DOY = 269) in the first growing season and by 27 September (DOY = 270) in the second growing season. The CO_2_ elevation, however, significantly affected the initiation, completion, and duration of leaf senescence (Table 1). Using 10% color change as an indication of leaf senescence initiation on pooled data across the photoperiod treatments, the leaf senescence under the elevated [CO_2_] started 23 days earlier but completed 7 days later than under the ambient (CO_2_; Figure 1D). The duration of the leaf senescence process was much longer under the elevated than ambient CO_2_, 65 days versus 25 days (Figure 1D). Furthermore, differences among different photoperiod treatments in the time course of leaf senescence tended to be greater under the elevated than the ambient CO_2_ (Figures 1A,B). Leaf senescence initiated earlier under the longer photoperiods corresponding to 55 and 58°N latitude than under the shorter photoperiods at 48 and 52°N latitude under the elevated [CO_2_], no such a difference occurred under the ambient (CO_2_; Table 1 and Figure 1C).

**TABLE 1:** Summary of analyses of variance (*F*-test statistics, *p* values, and degree of freedom) for the effects of [CO_2_], photoperiod regime, and their interactions on the timing of bud formation, leaf senescence (10, 50, and 90% based on leaf color change), duration of senescence (time difference between 10 and 90% color change), cold hardiness, and bud break.

Parameters	Growing season	[CO_2_]		Photoperiod		[CO_2_] × Photoperiodh	
		*F*	*p*	*F*	*p*	*F*	*p*
Bud formation	First	0.78	0.47	0.93	0.461	0.34	0.782
	Second	0.02	0.902	1.80	0.248	0.74	0.564
10% leaf color change	First	0.31	0.636	1.14	0.406	0.38	0.774
	Second	10.64	0.083	5.27	**0.041**	6.65	**0.025**
50% leaf color change	First	1.08	0.409	0.24	0.864	1.42	0.327
	Second	2.48	0.256	0.09	0.963	0.79	0.543
90% leaf color change	First	0.21	0.693	0.25	0.859	0.35	0.795
	Second	23.29	**0.040**	0.43	0.74	1.23	0.378
Duration of senescence	First	0.08	0.799	1.41	0.329	1.42	0.326
	Second	24.53	**0.038**	3.84	**0.076**	3.07	0.113
Cold hardiness	Fist	4.69	0.163	2.15	0.195	0.61	0.635
	Second	0.26	0.693	11.64	**0.009**	6.58	**0.038**
Bud break	Second	3.75	0.193	0.26	0.852	15.29	**0.003**
df			(df = 1)		(df = 3)		(df = 3)

*Seedlings were subjected to two different [CO_2_] (400 and 1000 μmol mol^–1^) and four photoperiod regimes (corresponding to 48, 52, 55, and 58°N latitude) for two growing seasons in environment-controlled greenhouses. Probabilities ≤ 0.05 are in bold.*

### Cold Hardiness

**FIGURE 2:**
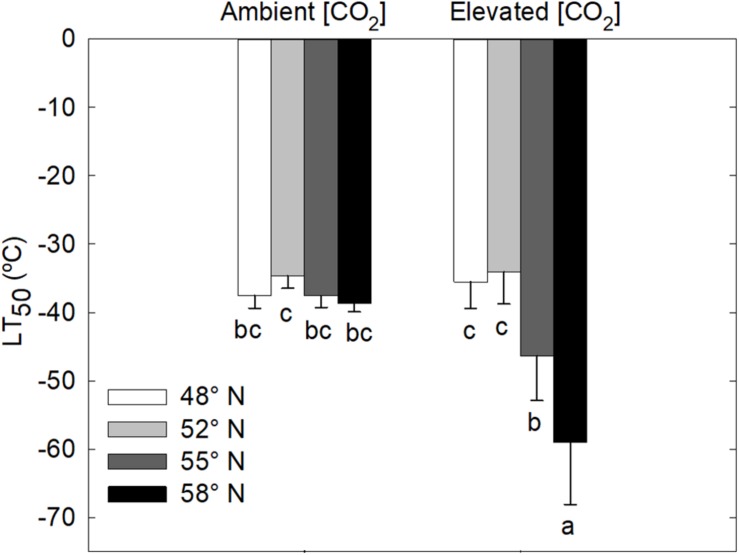
Freezing tolerance (LT_50_ = the lethal temperature for 50% tissue injury) of stem segments of white birch seedlings as affected by photoperiod regime and [CO_2_]. Each bar represents the mean ± SE of 8 seedlings. Different letters indicate that the means are significantly different from each other. See Figure 1 for more explanations.

LT_50_ was significantly affected by the [CO_2_] – photoperiod interaction in the second growing season but not in the first growing season (Table 1 and Figure 2). The CO_2_ elevation substantially increased the cold hardiness only at the two higher latitudes (55 and 58°N): the average LT_50_ was −60°C at 58°N and −46°C at 55°N while the average LT_50_ was less than −40 in all other treatment combinations (Figure 2). Furthermore, LT_50_ was significantly and substantially more negative at the two high than at the two low latitudes under the elevated CO_2_ while photoperiod had no significant effect on LT_50_ under the ambient CO_2_ (Figure 2). In other words, under the elevated CO_2_, the seedlings endured much colder freezing temperatures under the photoperiod regimes corresponding to the two higher than those corresponding to the two lower latitudes.

### Bud Break in the Spring

**FIGURE 3:**
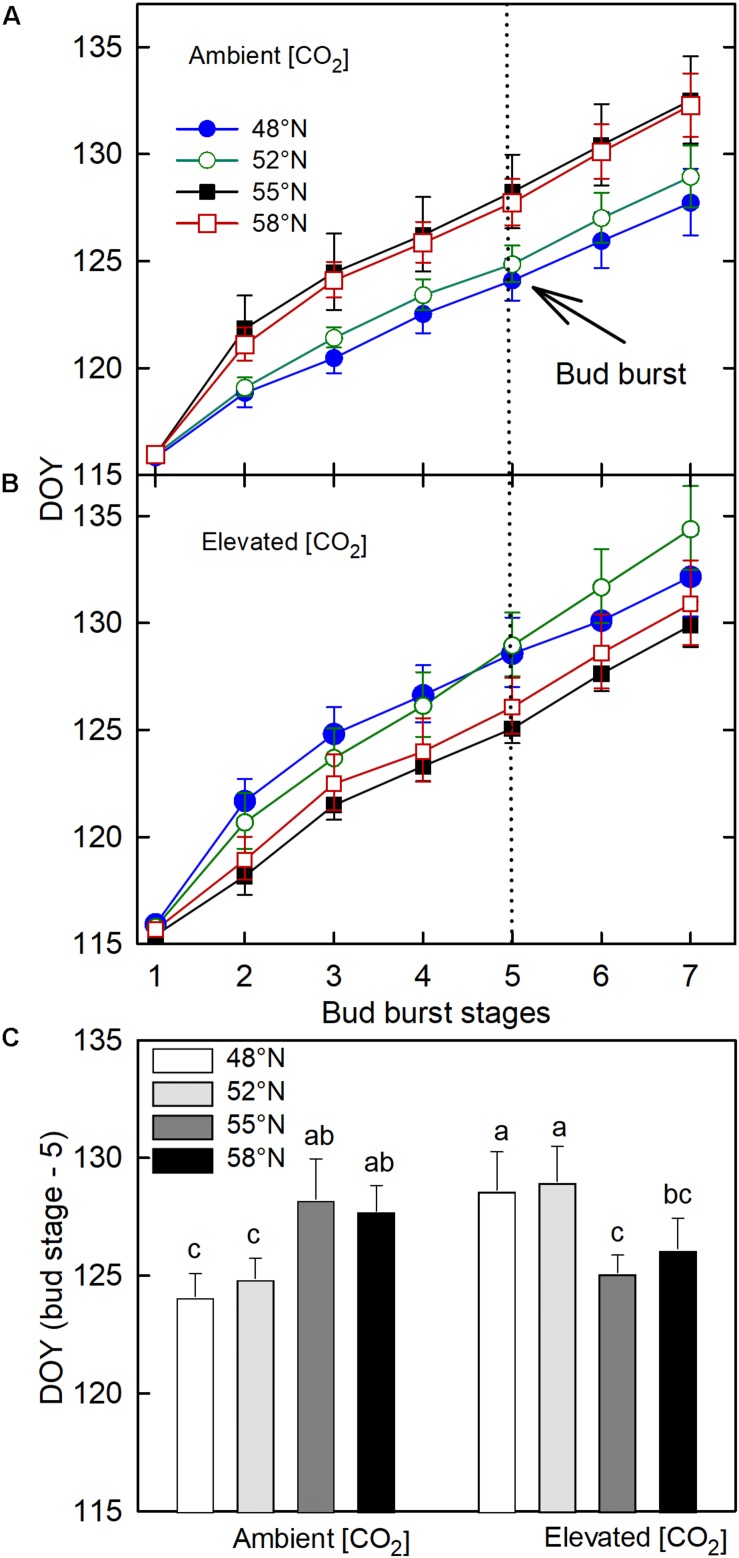
The effect of photoperiod regime on the progression of spring budburst under ambient **(A)** and elevated (CO_2_; **B)**. **(C)** the interactive effect of CO_2_ and photoperiod on the completion date (DOY = Stage – 5) of spring budburst with different letters within each CO_2_ and between ambient and elevated CO_2_ at a given photoperiod indicate significant difference from each other. The value of each point represents mean ± SE of 8 seedlings. See Figure 1 for more explanations.

The CO_2_ – photoperiod interaction significantly affected the number of days required to reach each phase of bud break (Table 1): under the ambient CO_2_, the seedlings grown under the photoperiod regimes of 55 and 58°N latitude broke their buds much slower than those grown under the two shorter photoperiod regimes (Figure 3A); under the elevated [CO_2_], however, the trend was the opposite (Figure 3B). The average date for reaching stage-5 of the bud burst in the two long photoperiods was 5 May (125 DOY) under elevated [CO_2_] and was 8 May (DOY = 128) under ambient (CO_2_; Figure 3C). However, no significant differences were found between the photoperiod regime of the seed origin and that at 52°N latitude or between 55 and 58°N (Figure 3C). Furthermore, the CO_2_ elevation significantly delayed budburst at the photoperiod regimes corresponding to the two lower latitudes (48 and 52°N), significantly advanced it at 55°N, but had no significant effect at 58°N (Figure 3C).

## Discussion

The most interesting finding of this study is that the CO_2_ elevation had different effects at different stages of leaf senescence. Elevated CO_2_ advanced the initiation of color change in the fall by an average of 23 days, but delayed the completion of leaf senescence, lengthening the process of leaf senescence. For deciduous trees, chlorophyll degradation is the first key stage of the leaf senescence process when resources from senescing leaves are resorbed and stored in the tree (Keskitalo et al., 2005). During the initial stage of leaf senescence, photosynthetic assimilates provide the energy needed for the dismantling process (Keskitalo et al., 2005). Therefore, any edaphic or environmental factor that accelerates leaf coloration can shorten the period of photosynthesis and reduce nutrient resorption (Richardson et al., 2010). In contrast, delays in the completion of leaf senescence could prolong the duration of carbon fixation and favor more completion resorption of nutrients (Richardson et al., 2010). However, it is unknown how much the delayed senescence can contribute to the net primary productivity and nutrient resorption in *Betulla* spp. (Goulden et al., 1996) or how much of the positive effect is offset by decreases in photosynthesis associated with the earlier start of leaf senescence. Any increase in carbon fixation after growth cessation can have a positive effect on bud development, winter storage, and spring leaf-out (Horwath et al., 1994). However, the delayed completion of leaf senescence may increase the risk of premature leaf-fall if a substantial drop in temperature occurs, which will halt the process of cold hardening and nutrient resorption, and affect the cold hardiness and growth in the following growing season (Way and Montgomery, 2015). While an earlier onset of leaf senescence under elevated [CO_2_] is commonly observed in deciduous trees (Körner et al., 2005), opposite results have also been reported (Godbold et al., 2014). CO_2_ elevation induced earlier onset of leaf senescence has been attributed to its indirect effect on the biochemistry and physiology of leaves (Jach et al., 2001), such as increases in starch and sugar content and decreases in nitrogen (Ainsworth and Long, 2004).

Another interesting finding is that the process of leaf senescence under the elevated [CO_2_] started earlier under the photoperiod regimes of the two high latitudes (55 and 58°N) than those at the two low latitudes (48 and 52°N) while no such differences occurred under the ambient [CO_2_]. The average threshold photoperiod for the initiation of leaf senescence for all four photoperiod treatments was 11:46 h under the ambient [CO_2_], whereas under the elevated [CO_2_], the average photoperiod was 13:46 h for the photoperiod treatments of 55 and 58°N latitude and 12:35 h for the treatments of 48 and 52°N latitude. While past studies have reported consistent relationships between photoperiod and timing of leaf senescence in deciduous trees (Velling, 1979; Fracheboud et al., 2009), our study shows that photoperiod can change the timing of autumnal leaf senescence only under elevated [CO_2_] and CO_2_ elevation can modify the relationship between leaf senescence and photoperiod. The earlier onset of leaf senescence may compromise the proper development of cold hardiness and buds. It may also reduce the tree’s ability to take advantage of the favorable environmental conditions late in the summer or early fall if trees migrate to higher latitudes under a changing climate in the future. In addition to environmental controls, spring phenology can also affect the timing of phenological processes in the fall (Fu et al., 2014; Keenan and Richardson, 2015). Nonetheless, such carryover effects were not significant in this current study (data not shown). Our results suggest that the interacting effects of CO_2_ elevation and photoperiod on the timing and duration of leaf senescence should be considered in studies or models on climate change-induced northward migration of boreal trees.

White birch seedlings in this study did not respond to treatments in the first growing season. In the second growing season, however, the seedlings grown under the elevated [CO_2_] and longer photoperiods tolerated much colder freezing temperatures. Under elevated [CO_2_], the LT_50_ was −46 and −60°C, respectively, for the photoperiod regimes associated with 55 and 58°N latitude while it was above −40°C for the photoperiod regimes associated with the two lower latitudes and there was no significant photoperiod effect under the ambient [CO_2_]. Wayne et al. (1998) have found that CO_2_ elevation enhanced the freezing tolerance of *B. allaghanensis* seedlings. The enhancement of freezing tolerance by CO_2_ elevations may be attributable to increases in the production of carbohydrates and cryoprotective sugars (Repo et al., 1996; Gandin et al., 2011). Furthermore, the prolonged process of leaf senescence in the combinations of elevated CO_2_ and longer photoperiods may indicate that those seedlings had a longer period of time to develop cold hardiness. Bigras and Bertrand (2006) have found that elevated [CO_2_] lead to an earlier start of fall phenological events and better frost tolerance in *Picea mariana* seedlings. However, it is not clear why the trees in this study did not respond to the treatments.

It is interesting to note that photoperiod regimes had opposite effects on the timing of spring budburst in white birch under the two different [CO_2_], and the effects of [CO_2_] also varied with photoperiod regimes. The spring budburst in seedlings grown in the photoperiod regimes at 55 and 58°N latitude occurred earlier than those grown under the photoperiod regimes at 48 and 52°N latitude under the elevated [CO_2_], but the trend was the opposite under the ambient [CO_2_]. Further, the CO_2_ elevation delayed the spring budburst at the two lower latitudes while it either advanced it or had no significant impact under the photoperiod regimes at the higher latitudes. Although temperature is believed to be the primary factor controlling spring budburst, exposure to longer photoperiods can reduce the required temperature sum for budburst (Heide, 1993a, b; Basler and Körner, 2014). However, in the present study, accelerated bud break at the longer photoperiods was apparent only under the elevated [CO_2_]. While most past studies find that CO_2_ elevations have no significant effects on the timing of bud burst or other spring phenological events in trees, some studies have reported that CO_2_ elevation can advance or delay spring phenology (Murray and Ceulemans, 1998). The effects of CO_2_ elevation and photoperiod on the timing of spring budburst may be related to the availability of soluble sugars (Oren et al., 1988). Since both CO_2_ elevation and longer photoperiod can increase the production and storage of soluble sugars (Gandin et al., 2011) and enhance freezing resistance (Repo et al., 1996), it is possible that their combination accelerates budburst in the spring. Although the earlier budburst may increase the risk of late frost damage if trees are not synchronized with the season transitions at the new location (Aitken and Hannerz, 2001), the simultaneous increase in cold hardiness in the treatment combination suggest that white birch can potentially have higher rates of growth at higher latitudes under future elevated [CO_2_] without increased risk of frost damages (Jach et al., 2001).

The present study emulated the seasonal changes in photoperiod associated with the latitude of the seed source and three latitudes north of the seed origin. However, we did not emulate the diurnal or seasonal changes in the quality of the light, i.e., the spectrum composition. The spectrum composition of the solar radiation at specific location changes with the time of day and season due to changes in sun angle. Some studies have indicated that the spectral composition of the light can affect plant phenology, particularly the red to far-red light (R:FR; Heide, 1993a; Olsen et al., 1997). A reduction in the R:FR ratio in the early morning and/or evening hours has been shown to control growth cessation in aspen in the fall (Olsen and Junttila, 2002) and expedite spring bud burst in *Betula pendula* (Linkosalo and Lechowicz, 2006). Therefore, this and similar studies can only shed light on the effect of changes in day-length but not changes in the quality of light. However, further studies that combine all aspects of the seasonal light regime associated with the change in latitude/photoperiod during tree migration in response to climate change are warranty.

It is believed that migration to higher latitudes will lead to longer growing seasons for trees because the threshold photoperiod triggering leaf senescence and cold hardening in the fall will arrive later (Thomas and Vince-Prue, 1997). The results of this study suggest that the matter is more complicated. The combination of longer photoperiods and elevated [CO_2_] led to an earlier onset and longer-duration of senescence in the fall and earlier budburst in the spring in white birch. Therefore, the growing season was extended at both ends of the season. Furthermore, the treatment combination also resulted in greater cold hardiness in the seedlings. However, the photoperiod effects were quite different under the ambient [CO_2_]. Our results suggest that it is extremely important to consider the complex interactions of [CO_2_] and photoperiod in planning latitudinal seed transfers and in predicting the migration of boreal trees in response to climate change.

## Data Availability Statement

The raw data supporting the conclusions of this manuscript will be made available by the authors, without undue reservation, to any qualified researcher.

## Author Contributions

BT performed the research and participated in all phases of the study. Q-LD and SI contributed to experimental design, data interpretation, manuscript writing, and discussion of ideas. All authors read and approved the final version of the manuscript.

## Conflict of Interest

The authors declare that the research was conducted in the absence of any commercial or financial relationships that could be construed as a potential conflict of interest.

## Footnotes

1 http://www.esrl.noaa.gov/gmd/ccgg/trends/index.html
